# Repurposing Peroxisome Proliferator-Activated Receptor Agonists in Neurological and Psychiatric Disorders

**DOI:** 10.3390/ph14101025

**Published:** 2021-10-08

**Authors:** Claudia Sagheddu, Miriam Melis, Anna Lisa Muntoni, Marco Pistis

**Affiliations:** 1 Department of Biomedical Sciences, Division of Neuroscience and Clinical Pharmacology, University of Cagliari, 09042 Monserrato, Italy; claudiasagheddu@unica.it (C.S.); myriam@unica.it (M.M.); 2 Neuroscience Institute, National Research Council of Italy (CNR), Section of Cagliari, 09042 Monserrato, Italy; amunto@unica.it

**Keywords:** PPARs, pioglitazone, fenofibrate, fibrates, *N*-acylethanolamines, neuroinflammation

## Abstract

Common pathophysiological mechanisms have emerged for different neurological and neuropsychiatric conditions. In particular, mechanisms of oxidative stress, immuno-inflammation, and altered metabolic pathways converge and cause neuronal and non-neuronal maladaptative phenomena, which underlie multifaceted brain disorders. The peroxisome proliferator-activated receptors (PPARs) are nuclear receptors modulating, among others, anti-inflammatory and neuroprotective genes in diverse tissues. Both endogenous and synthetic PPAR agonists are approved treatments for metabolic and systemic disorders, such as diabetes, fatty liver disease, and dyslipidemia(s), showing high tolerability and safety profiles. Considering that some PPAR-acting drugs permeate through the blood–brain barrier, the possibility to extend their scope from the periphery to central nervous system has gained interest in recent years. Here, we review preclinical and clinical evidence that PPARs possibly exert a neuroprotective role, thereby providing a rationale for repurposing PPAR-targeting drugs to counteract several diseases affecting the central nervous system.

## 1. Introduction

Over the past decade, evidence has shown converging mechanisms for different brain disorders whereby inflammation and cellular metabolic imbalance have emerged at different stages in neuropathological processes. Within the wide spectrum of neuropsychiatric disorders, finding overlapping substrates is key to managing the therapeutics and efficiently counteracting the clinical outcome. In this view, peroxisome proliferator-activated receptors (PPARs) are considered multi-purpose molecular targets. PPARs have been involved in medical conditions ranging from hepatocellular to myocardial diseases, and have been successfully targeted for drug development and pharmacological treatment. Evidence of the participation of PPARs in brain disorders is relatively recent, although remarkable. Genome-wide association studies, together with computational approach, suggest new therapeutic opportunities for PPAR agonists, which were already used for decades to treat different peripheral pathologies. Hence, the repurposing of existing drugs against central nervous system (CNS) diseases is prominent as a rapid and rationally “safe” approach, and is also promising for multiple organ dysfunction syndromes, as we will review and discuss in the following sections.

## 2. PPARs: From the Periphery to CNS

PPARs are intranuclear receptors, which serve as ligand-activated transcription factors [1,2]. Their name comes from the original observation that chronic activation of these receptors causes hepatic peroxisome proliferation in rodents [3]. Although this effect is not observed in humans and is limited to PPARα, the name remains. Like other nuclear receptors, PPARs, once activated, bind to consensus DNA sequences to regulate gene expression through transcriptional co-activation [4,5,6]. The PPAR subfamily comprises three isoforms: PPARα, PPARβ/δ and PPARγ, which share a structural homology [1,2,7,8]. PPARs are activated by small, lipophilic molecules and, in the cytoplasm, form heterodimers with the retinoid X receptor-α (RXR) to regulate gene expression [9].

The activated protein-DNA complex, PPAR/RXR, targets the peroxisome proliferator response element (PPRE) within the gene promoter [1,10], eliciting intracellular molecular cascades [10].

PPARs are recognized to regulate the expression of several target genes in both the periphery and the CNS. These are primarily implicated in energy regulation, lipid and glucose metabolism, and in anti-inflammatory processes. Considering their pleiotropic functions, the investigation of pathological deregulation of PPARs, together with their potential pharmacological activation, is a topic of increasing interest in both preclinical and clinical research. The implications of PPARs as lipid sensors are well known in whole-body adipogenesis and hepatic homeostasis, so that their impairment is associated with progressive liver fibrosis and steatosis [11,12,13]. PPARs have also been involved in regulating mitochondrial β-oxidation and cellular growth [14], with the β/δ isotype being particularly considered as a potential therapeutic target for metabolic syndromes [15,16,17].

Among the endogenous agonists, the fatty acid ethanolamides, also known as *N*-acylethanolamines (NAEs), show a high affinity. These molecules are long-chain fatty acids [18,19] that are ubiquitously found in animal tissues [20], in both the periphery and the brain. The most-studied NAEs are arachidonoylethanolamide (anandamide, AEA), palmitoylethanolamide (PEA) and oleoylethanolamide (OEA). Other, less-characterized NAEs are stearoylethanolamide (SEA) and linoleoylethanolamide (LEA) [21,22]. AEA, the first endocannabinoid to be identified [23], is the only NAE that binds to cannabinoid type 1 (CB1) and type 2 (CB2) receptors with high affinity. PEA and OEA were known for many years, but their role, as well as that of other NAEs, in the CNS, was only characterized in the last two decades [24].

PPARα agonists such as fibrates have long been clinically used to treat hypertriglyceridemia, whereas PPARγ agonists, such as thiazolidinediones, are approved for type-2 diabetes (T2D) [25,26,27]. Recent findings show the impact of PPARs in cancer development, although whether this is a direct influence or a consequence of their target gene transcription has not been elucidated [26,28,29].

In the CNS, PPAR mRNA and protein are ubiquitously expressed, suggesting that these receptors are involved in the regulation of neuron and glial cell metabolism and energy balance. Different brain disorders, including neurological, neuropsychiatric and neurodegenerative diseases, share general etiopathogenic factors such as an altered cellular metabolism, modifications in synaptic/electrical activity and concurrent inflammatory processes. Remarkably, PPARs regulate genes that are involved in all of these components. This suggests that the agonists for different isotypes of PPAR could be attractive pharmacological tools that might regulate different pathological mechanisms at once. Their potential contribution against inflammation, pain, demyelination, and epileptic seizures [30,31] is emerging; preventive neuroprotection in the early stages of degenerative disorders would also be desirable [32]. Increasing evidence shows the pivotal role of PPARs in the whole organism and overlapping physiopathological processes for different disorders; thus, the repositioning of the already marketed PPAR-acting medications in brain disorders appears attractive. Moreover, given the partial overlap in genes modulated by PPARα and -γ, newly developed co-activators, such as the dual-acting agonist tesaglitazar, deserve special mention for future clinical applications [33,34].

## 3. The Pleiotropic Mechanism of Action of PPARs: Transcriptional Effects and Rapid Synaptic Regulation

PPARs display isoform-specific tissue expression, as well as a plethora of endogenous ligands, which show different binding affinities. Inducing/suppressing control of several genes ultimately leads to a multifaceted impact, in both physiological and pathological conditions. For instance, pan-PPAR targeting would regulate the overall energy balance [13]. Nevertheless, PPARα would mostly influence the fatty acid metabolism and promote low triglyceride levels, while PPARγ would mainly influence lipid biosynthesis/adipogenesis, glucose metabolism and intracellular insulin signalling, and PPARβ/δ would operate on fatty acid oxidation and blood glucose [17,26,35].

In the brain, activation of PPARs occurs in genetic mechanisms at different levels, ranging from basal cellular neurobiology to complex cognitive processes. Hence, PPARs positively or negatively modulate the genes that are implicated in sleep/circadian rhythm, feeding behaviors, neuroinflammatory and degenerative processes, leading to overall neuroprotective effects [32,36]. In this regard, PPARγ, which is the most abundant isoform in microglia, suppresses the immunoinflammatory response by regulating genes for the biosynthesis of cytokines, prostaglandins and nitric oxide, as well as inducing the apoptosis of reactive microglial cells [37]. PPARα modulates the synthesis of proteins involved in neuroprotection and regulates synaptic signaling. At present, the -β/δ is the least-characterized isoform in the CNS, although it is only highly expressed in neuronal cell types [38]. However, a relevant recent study found PPARβ/δ to be repressed by the mutant form of huntingtin (HTT) in Huntington’s disease (HD), while its activation reverted transcriptional alterations associated with neurodegeneration, motor impairments and mitochondrial abnormalities in experimental models of HD [39,40]. According to the emerging evidence, the repurposing of PPAR agonists is increasingly promising in several human brain disorders.

In addition to the multiple, even opposing, genomic actions of PPARs, rapid effects have also been described. Non-liganded PPARβ/δ negatively regulates the -α and -γ downstream signaling, suggesting cross-talk amongst isoforms when expressed in the same cell type [41]. This mutual tuning prompts possible implications concerning PPAR-based therapeutical approaches. 

PPARγ has been shown to affect, among others, platelet functionality. In different cells, PPARγ represses β-catenin signaling and/or triggers mitogen-activated protein kinase (MAPK) pathways. Specifically, PPARγ-dependent phosphorylation was observed over the classical extracellular signal-regulated kinase tipe1/2 (MAPK ERK1/2) in tumoral cell lines, in contrast with the dephosphorylation of myosin phosphatase in aortic smooth myocytes. Finally, PPARα also exhibits non-genomic effects. For instance, agonists for α isotype reduce glucose-induced intracellular Ca^2+^ concentration and insulin secretion in pancreatic β cells and regulate β subunit phosphorylation in nicotinic acetylcholine (ACh) receptors expressed by midbrain dopamine neurons, modulating their electrical activity in turn. This mechanism has particular relevance for several dopamine-associated neurological and neuropsychiatric conditions [24,42]. The peculiar dual-genomic and non-genomic pathway that follows the activation of PPARα warrants further investigation and suggests that these nuclear receptors are a promising therapeutic target in diverse psychiatric and neurological illnesses [43,44].

Neurodevelopmental disorders such as autism and schizophrenia [45,46,47,48], as well as mood disorders [43,49,50], have been shown to exhibit a disruption of neuroimmune functions. Thus, it is particularly tempting to repurpose PPAR agonists for neurological and psychiatric disorders, as detailed in the following sections.

## 4. PPAR Agonists in Psychiatric Disorders

PPARα seems to be critically involved in the pathophysiology of schizophrenia [44,51,52]. The evidence for this derives from preclinical studies with the maternal immune activation (MIA) model or the postnatal lesional model and from clinical investigations into the association of schizophrenia with PPARα genes. In the MIA model, an inflammatory insult is evoked at a precise stage of pregnancy. Consequently, this insult disrupts brain development in offspring, which shows behavioral, neurophysiological and neurochemical imbalances. The PPARα agonist fenofibrate, which is approved for the treatment of hypercholesterolemia and hypertriglyceridemia, was shown to attenuate behavioral disruption and dopaminergic dysfunction in MIA offspring [53,54]. Although the precise mechanism is still under investigation, it is hypothesized that PPARα activation negatively regulates the maternal inflammatory response by dampening nuclear factor kappa-light-chain-enhancer of activated B cells (NFκB) signaling, activator protein 1 (AP-1) [55], tumor necrosis factor-α (TNF-α) [56], as well as inhibiting the production of cytokines and interferons. Fenofibrate was also evaluated in a neurodevelopmental rat model of schizophrenia consisting of postnatal kainic acid injection. In this model, fenofibrate attenuated the disruption of pre-pulse inhibition, a measure of sensorimotor gating functions whose deficits accompany a psychotic-like phenotype in young adulthood [57]. The gene encoding for PPARα (PPARA) was found to be downregulated in hair follicle cells from schizophrenia patients [51], further showing an association between PPARα and this psychiatric condition [52].

Consistent with the notion that schizophrenia displays a pro-inflammatory phenotype [45,46,47], genes involved in inflammation have been found to be affected, including decreased PPARα mRNA levels and increased IL-6 and TNFα. Additionally, a study on a Croatian population investigated whether a functional L162V polymorphism in the PPARα gene was also associated with schizophrenia risk [58]. Remarkably, the PPARα-L162V polymorphism impacts the clinical manifestation (i.e., severity) of the illness, plasma lipid concentrations, and the risk of addiction to tobacco (i.e., nicotine) in female patients. Nonetheless, an association with schizophrenia risk was not reported [58,59].

Rosiglitazone, a PPARγ agonist approved for T2D, has been proposed to improve the cognitive symptoms of schizophrenia due to its ability to induce the expression of BDNF [60]. However, in a pilot study by Yi et al. [61], rosiglitazone administration had no significant beneficial effect on the cognitive performance of patients with schizophrenia, who were also administered with clozapine. Conversely, results from Watson et al. [62] showed improved cognitive tasks, at least in Alzheimer’s Disease (AD)-affected patients treated with rosiglitazone. The contrasting results are not surprising, given the differences in study populations, dosing, and treatment duration [61]. On the other hand, pioglitazone (another PPARγ agonist approved for T2D) showed a positive effect. Smith et al. [63] demonstrated that pioglitazone is efficacious to treat glucose and lipid abnormalities in schizophrenic patients, and reduces their symptoms, as measured by the positive and negative syndrome scale (PANSS). Based on the high prevalence of diabetes in patients with schizophrenia [64], pioglitazone, concomitant with risperidone, appeared to be beneficial for counteracting negative symptoms in schizophrenia [65], such as lack of concentration, loss of interest, and social withdrawal.

The converging evidence suggests a reciprocal modulation of depressive states and inflammation [50,66,67,68]. Notably, stress plays a critical role in the expression of depressive symptoms, being associated with alterations in immunological and neuroendocrine responses, as well as circuit changes perpetuating depressive moods [68]. Furthermore, imbalances in cholinergic systems have long been hypothesized to be crucially involved in the pathophysiology of depression [69,70]. In this scenario, the observation that activation of PPARα is engaged by dopamine cells as a self-regulatory mechanism in response to a hypercholinergic drive [71] makes it a potential target in the treatment of depressive states. Accordingly, in rodents, the acute direct and indirect stimulation of PPARα results in an effective anti-depressant-like activity [71,72,73]. Chronic administration of either synthetic or natural PPARα ligands has been shown to prevent and relieve depressive behaviors resulting from chronic stress exposure [49,72,73,74], as well as restoring dopamine system function [49] and hippocampal BDNF signaling cascade [73], which are key to the pathogenesis of mood disorders [75,76]. Notably, not only is the PPARα-dependent enhancement of hippocampal BDNF signaling and neurogenesis involved in the pathophysiology of depression, it is also involved in the mechanism of action of the antidepressant fluoxetine [77]. These effects add to the many other actions triggered by these receptors, including the increased biosynthesis of neuroactive steroids, which exert antidepressant activity [78]. This is not unexpected, as PPARα is a nuclear receptor at the crossroads of many signaling pathways and, as mentioned above, acts via genomic and non-genomic actions. Hence, the clinical evidence suggesting that the activation of these receptors by synthetic agonists is effective in diverse depressive states [43,78,79], along with their safe pharmacological profile, further demonstrates that it may be a promising and feasible antidepressant target.

PPARγ agonists (i.e., pioglitazone, rosiglitazone) have proven their efficacy as antidepressants in animal models [80,81], as well as in patients that also presented insulin resistance [82,83,84]. Moreover, depressive symptom levels have been correlated with molecular alterations, such as the dysregulation of interleukin (IL)-6 levels [84], a further anti-inflammatory effect of PPARγ agonists.

The localization of PPAR isoforms in brain regions involved in the neurobiology of substance-use disorders (SUDs) [85], together with their broadly investigated influences on mesocorticolimbic dopamine system neurophysiology [24,42,86,87], highlights PPAR potential in this psychiatric condition [88,89]. Indeed, accumulating evidence suggests that neuroinflammation contributes to imbalanced reward/aversion circuits, leading to addictive behaviors. The stages of drug dependence, oversimplified as abuse/binge–high–withdrawal/compulsion for reuse–relapse (see [90] and [91]), suggest multiple therapeutical timepoints for successful treatment. The PPAR signaling cascade and their metabolic machinery are being considered as an innovative target, among others, for preclinical investigations into the development of therapies against alcohol [92,93,94,95,96,97], tobacco [98,99,100,101,102,103,104,105,106], opioid [107,108,109], and psychostimulant [110,111] dependence. Hence, the repurposing of PPAR-based medications is of increasing interest in this area of neuropsychiatry [111,112,113], although controversial results in humans [114,115] suggest that cautiousness is required. Table 1 summarizes the preclinical and clinical studies aiming to repurpose PPARα and PPARγ agonists in the treatment of neurological and psychiatric diseases.

## 5. PPAR Agonists in Neurological Disorders

Given their multifaceted pharmacological properties (anti-inflammatory, neurotrophic/neuroprotective), PPAR pathways have also attracted considerable attention as potential therapeutic targets, as well as risk factors, in various neurological disorders, including AD, multiple sclerosis (MS), Parkinson’s Disease (PD), and epilepsy [43,44,79,132,133] (Table 1). PPARs appear to play a direct role in the pathophysiology of AD [134,135,136], the leading cause of dementia in the elderly. From a neuropathological perspective, PPARα has been implicated in the etiopathogenesis of AD, particularly in the upstream homeostasis of amyloid precursor protein (APP) [135]. The expression levels of PPARα and β/δ are significantly reduced, while PPARγ is selectively increased in AD brains, suggesting that a dysfunctional PPAR system might contribute to AD’s onset and progression. Accordingly, not only have several studies reported promising effects of different PPAR agonists in experimental models, but these effects have also been reported in AD patients [79,134,135,136]. Among the three receptor isotypes, the PPARγ is the first and the most extensively studied. Hence, initial preclinical and clinical evidence showed that PPARγ modulators improve learning and memory by reducing microglial activation and Aβ plaques, in both humans [62,126,137,138,139] and an AD mouse model [43,125]. However, in clinical trials, neither rosiglitazone or pioglitazone yielded meaningful outcomes [79,128,129]. Several in vitro and in vivo studies point to PPARα as an emerging therapeutic target for AD due to the ability of both natural (e.g., PEA) and synthetic agonists (e.g., fibrates) to modulate AD pathogenetic mechanisms and progression [136,140]. Scuderi et al. revealed that PEA’s anti-inflammatory properties accounted for a PPARα-dependent reduction in astrogliosis, pro-inflammatory signals and neuronal loss [141,142,143,144,145].

PPARα has been shown in preclinical models to be key for the neuroprotective and memory-rescuing effects of PEA [144,146]. It has consistently been shown that administration of gemfibrozil or permafibrate in a well-characterized model of familial AD (5XFAD transgenic mice co-expressing five mutations of familial AD and characterized by rapid brain amyloidosis) decreases amyloid plaque deposition, microgliosis and astrogliosis in the hippocampus and cortex, and is associated with a significant improvement in spatial learning, memory and hippocampal plasticity [122,123]. Luo and colleagues demonstrated that either gemfibrozil or Wy14643 reverse not only memory deficits, but also amyloid plaque pathology and anxiety in the APP-PSEN1ΔE9 model (co-expressing the Swedish APP mutation and exon 9 deletion of the PSEN1 gene) via a PPARα-dependent enhancement of autophagosome biogenesis [124]. Investigation of the β/δ isoform in the pathophysiology of AD is in the early stages [36]. However, a phase II clinical trial for the T3D-959 dual-agonist for the -β/δ and -γ isoforms [147] showed promising results for further studies in AD patients. Finally, the co-activator-1α (PGC1α), a PPARs’ transcriptional regulator, represents a possible therapeutic target. PGC1α protein levels were found to be reduced in the Tg2576 mouse model (that overexpresses a mutant form of APP with the Swedish mutation KM670/671NL, which increases Aβ levels and amyloid plaques) [148], and the mRNA in the brain of AD patients correlated with the progression of clinical dementia [149]. Accordingly, the polyphenol resveratrol has proven to be neuroprotective through the modulation of PGC1α [150,151]. Collectively, these findings support that, in AD, repurposing the thiazolidinediones as PGC1α/PPARγ-activating drugs warrants further investigations (see [152] for an extensive review).

Due to their role in the regulation of neuroinflammation and immune responses, PPAR dysfunction might also be involved in the mechanisms underlying MS [153,154], the most common disabling neurological condition among young adults. MS patients show decreased PPARγ expression [155] as well as reduced levels of NAEs (i.e., OEA, PEA, AEA) [156]. Of note, the latter tend to increase during MS clinical exacerbation, probably because NAEs act as endogenous neuroprotective molecules [156]. This is in accordance with the possible compensatory mechanism induced by increased levels of PEA in the CNS, as indexed by the reduced progression of neurodegenerative markers in two different mouse models of chronic MS

In the same MS chronic models, PEA administration reduced spasticity and motor disability, together with an anti-inflammatory effect [157,158]. PEA also reduced the expression of inflammatory cytokines in the myelin oligodendrocyte glycoprotein-induced experimental autoimmune encephalomyelitis (MOG-EAE) mice, an effect accompanied by decreased demyelination and axonal damage [159]. Similarly, the PPARγ agonist troglitazone attenuates inflammation and ameliorates EAE-related signs in mice [131]. Unfortunately, clinical data on the potential therapeutic use of PPARγ agonists are limited [43,79], although both drugs have proven efficacy in reducing lesion burden. The need to upscale these trials is, therefore, critically relevant.

Concerning PD, the most prevalent neurodegenerative movement disorder [160], a large body of evidence supports the protective/neurotrophic properties of different PPAR ligands [44]. OEA attenuates behavioral symptoms and exerts neuroprotection on the nigrostriatal system in an experimental model via a PPARα-dependent mechanism [161,162]. In addition, chronic treatment with PEA counteracts 1-methyl-4-phenyl-1,2,3,6-tetrahydropyridine (MPTP)-induced glial cell activation, loss of nigral dopamine neurons, behavioral impairments, and motor dysfunctions, while the genetic ablation of PPARα exacerbates MPTP systemic toxicity [163]. Consistently, Barbiero et al. demonstrated the ability of the synthetic PPARα agonist fenofibrate to protect against the detrimental effects of MPTP in a rat model of PD [116,117]. The observation that both PEA and fenofibrate exert a neuroprotective effect after the onset of the pathology is remarkable, as disease-modifying drugs administered during disease evolution may delay the progression.

This is particularly significant in PD, as possible pharmacotherapeutic interventions are limited once the dopamine system has degenerated and subsequent symptoms have appeared [44,163].

Similarly, PPARγ ligands have been reported to mitigate PD progression in preclinical settings [43,164,165,166]. For instance, in different MPTP models, pioglitazone reduced microglial activation and iNOS-positive cells and expression of the monoamine oxidase B gene, which are considered protective against neurotoxicity, whereas rosiglitazone counteracted dopamine neuron loss and prevented olfactory and motor dysfunctions [127,130,166]. Accordingly, the PPARγ agonist, MDG548, and the PPARα/γ dual agonist, MHY908, exert neuroprotective effects by boosting phagocytosis and anti-inflammatory cytokines production, and by reducing microglial activation and neuroinflammation in MPTP mouse models of PD, thus attenuating neuronal damage and motor deficit [167,168]. When combined, these findings further support the involvement of activated PPARγ in microglial function, phagocytosis and neuroinflammation [43]. It is also worth mentioning the interaction between PPARγ and PGC-1α, which has recently proven to be neuroprotective. On the other hand, the results of a study [169] in which the PPARγ agonist pioglitazone was used in an attempt to modify disease progression in PD did not support the initiation of further trials with this drug (see also [44,170]).

PPAR modulation also represents a promising pharmacological approach against a common and prevalent neurological disorder, that is epilepsy, which, in its different manifestations, affects up to 2% of the global population [171].

Selective PPARα agonists have been proven to raise seizure thresholds in animal models of epilepsy, suggesting their possible repurposing in seizure management in patients [172]. PEA, for example, displayed anticonvulsant effects in mice [173] and showed antiepileptic actions in kindled rats [174] as well as in a genetic model of absence epilepsy [175]. Next, a study by Saha et al. [121] reported bezafibrate as being effective in the pentylentetrazole (PTZ)-induced kindling seizure model, and in reducing hippocampal cell loss in rat brain [121]. Likewise, the synthetic PPARα ligand fenofibrate and the ketogenic diet (KD) displayed the same efficacy as an anticonvulsant in adult rats, by decreasing PTZ-induced seizures and by extending the latency to the onset of epileptic symptoms induced by lithium-pilocarpine [118]. 

Moreover, acute and chronic fenofibrate is protective against nicotine-evoked epileptic manifestation and synchronization in the frontal cortex [119]. Lately, PPARα agonists have been proposed as a novel disease-modifying target for sleep-related hypermotor epilepsy (SHE), formerly referred to as nocturnal frontal lobe epilepsy (NFLE), idiopathic epilepsy with an autosomal inherited component, based on the effectiveness of fenofibrate in an NFLE mouse model and as an adjunctive therapy in pharmacoresistant NFLE patients [120]. The evidence also supports the repurposing of PPARγ agonists in epilepsy, given their neuroprotective and anti-seizure properties, with this isoform being involved in the KD mechanism of action [176], suggesting the repositioning of pioglitazone [177] and rosiglitazone [178,179,180] as therapeutic adjuvants for severe, refractory epilepsy.

## 6. Concluding Remarks

Since the advent of endocannabinoid system research, additional lipid signaling molecules have been found to play a role in brain functions. As they are ubiquitously expressed, PPARs are the new widgets in the pharmacologists’ toolkit. PPAR agonists are not miracle drugs, but their effects on lipid and glucose metabolism might affect the pathophysiological mechanisms of multifaceted psychiatric and neurological diseases. It is intriguing to speculate that one possible common denominator of all the disorders is the disruption of the neuronal and glial metabolism [181], which can be effectively modulated by PPARs.

Repurposing drugs by reprofiling their initial therapeutic indication, along with designing novel compounds with the same biological target, is pivotal to better understanding the pathophysiological underpinnings of brain disorders, but it is also paving new roads for their treatment.

## Figures and Tables

**Table 1:** This table summarizes the preclinical and clinical studies aiming to repurpose PPARα and PARγ agonists in psychiatric and neurological disorders. Further details can be found in the text.

	Preclinical or Clinical Study	Disease	Animal Model or Patients	Outcome	References
**PPARα agonist**					
**Fenofibrate**	preclinical	Schizophrenia	Rat: maternal immune activation	Attenuation of behavioral disruption and dopaminergic dysfunction	[53,54]
			Rat: postnatal kainic lesion	Attenuation of disruption of pre-pulse inhibition	[57]
		Depression	Rat: chronic stress	Antidepressant-like effect	[49]
		Parkinson’s Disease	Rat: MPTP model	Protection from neurotoxicity	[116,117]
		Epilepsy	Pharmacologically induced seizures or genetic models	Reduction in seizures	[118,118]
	clinical	Epilepsy	Sleep-related hypermotor epilepsy	Reduction in seizures	[119]
**Bezafibrate**	preclinical	Epilepsy	Pentylenetetrazole-Induced Kindling Seizure	Reduction in seizures	[121]
**Clofibrate**	preclinical	Nicotine Dependence	Nicotine self-administration in rats and monkeys	Blockade of nicotine self-administration and conditioned place preference	[105]
**Gemfibrozil**	preclinical	Alzheimer’s Disease	Transgenic AD mice	Decreases amyloid plaque deposition, astrogliosis; improves spatial learning, memory, and hippocampal plasticity	[122,123,124]
	clinical	Nicotine Dependence	Smokers	No efficacy	[115]
**PPARγ agonist**					
**Rosiglitazone**	preclinical	Diabetes-Induced Cognitive Decline	Mice model (diabetic mice)	Induction of BDNF expression	[60]
	clinical	Schizophrenia	Schizophrenic patients	no significant benefits on cognition	[61]
	preclinical	Depression	Rat: forced swimMouse: tail suspension tests	Antidepressant-like effect	[80]
	clinical		Depressed patients with insulin resistance	Effective as adjunctive treatment	[82]
	preclinical	Alzheimer’s Disease	Rat and mice models of Alzheimer’s disease	Improvement in learning and memory, Reduction in microglial activation and Aβ plaques	[43,125]
	clinical		Patients with Alzheimer’s disease	Improvement in cognitive abilities or no efficacy	[62,126]
	preclinical	Parkinson’s Disease	Rodent: MPTP model	Protection from neurotoxicity	[127]
**Pioglitazone**	clinical	Schizophrenia	Schizophrenic patients with metabolic syndrome	Treatment of glucose and lipid abnormalities in schizophrenic patients, and symptom reductions	[65]
	preclinical	Depression	Mouse: forced swimming test	Antidepressant-like effect	[81]
	clinical		Patients with moderate-to-severe major depressive disorder	Effective as adjunctive short-term treatment	[83]
	preclinical	Alzheimer’ s Disease	Mouse models of Alzheimer’s disease	Improvement in learning and memory; reduction in microglial activation and Aβ plaques	[43,125]
	clinical		Alzheimer’s disease patients	No efficacy	[128,129]
	preclinical	Alcohol Use Disorder	Rat models of alcoholism (alcohol preferring rats, binge alcohol drinking)	Reduction in alcohol seeking. Protection against neuronal and cognitive degeneration elicited by binge alcohol exposure	[95,96,97]
	clinical		Alcohol-dependent patients	No efficacy	[114]
	preclinical	Opioid Use Disorder	Morphine-dependent mice	Attenuation of morphine withdrawal symptoms, of reinstatement of heroin seeking and of heroin-induced reinstatement.	[107]
	clinical		Heroin users or nondependent prescription opioid abusers	Failure to alter the reinforcing or positive subjective effects of heroin. Reduction in heroin craving and anxiety. Failure to alter the abuse liability of oxycodone	[108,109]
	preclinical	Parkinson’s Disease	Rodent: MPTP model	Protection from neurotoxicity	[127,130]
**Troglitazone**	preclinical	Multiple Sclerosis	Mouse: experimental autoimmune encephalitis	Anti-inflammatory effects	[131]

## Data Availability

No new data were created or analyzed in this study. Data sharing is not applicable to this article.
